# Pre- and Post-partum Concentrations of Interleukin 1α, Interleukin 8, and α1-Acid Glycoprotein in Vaginal Fornix and Endometrium of Dairy Cows With Clinical Cervicitis

**DOI:** 10.3389/fvets.2020.605773

**Published:** 2021-02-02

**Authors:** Darío A. Vallejo-Timarán, Ali Bazzazan, Mariela Segura, Nelson E. Prieto-Cárdenas, Rejean C. Lefebvre

**Affiliations:** ^1^One Health and Veterinary, Innovative Research and Development (OHVRI) Research Group, School of Veterinary Medicine, University of Antioquia, Medellín, Colombia; ^2^Department of Biomedicine, Faculty of Veterinary Medicine, University of Montreal, Saint-Hyacinthe, QC, Canada; ^3^Department of Clinical Sciences, Faculty of Veterinary Medicine, University of Montreal, Saint-Hyacinthe, QC, Canada

**Keywords:** innate immunity, vaginal fornix, cervicitis, endometritis, inflammation, dairy cow, postpartum, uterine diseases

## Abstract

Innate immunity is the principal sensor responsible of the local immune response to control mucosal bacterial contamination of the reproductive tract after parturition, triggering a pro-inflammatory process in the mucosa of the uterus, the vaginal and the cervix. However, knowledge about the inflammation process and outcome of the cervix in dairy cows is scarce even though it plays an important anatomic and functional role between the vagina and the uterus. The objective of the present study was to describe the cellular and humoral local innate immune response during clinical cervicitis (CC) in the uterus and vaginal fornix in pre- and post-partum periods of dairy cows. A retrospective descriptive study was performed involving 26 animals, characterized as clinical cervicitis cows (*n* = 19) and healthy cows (*n* = 7). Blood and mucus of the different compartments of the genital tract were sampled and records of the cows' genital exam were performed four times: −1 w (day −7 ± 2, prepartum), +1 w (day +7 ± 4), +3 w (day +21 ± 4) and +5 w (day +35 ± 4) postpartum. Clinical cervicitis was defined as cows exhibiting a cervix grade−2 and healthy cows were defined as a cow clinically normal with a grade-0 cervix at time +5 w. Blood white cell count, vaginal fornix and endometrial neutrophils percentage, and the concentrations of interleukin 1α (IL1), interleukin 8 (IL8), and α1-acid glycoprotein (AGP) in mucus were determined. The results showed that 23% of the cows were categorized as CC at time +5 w. Cases of CC with purulent vaginal discharge or subclinical endometritis shown the highest cytokine production. At +3 w, IL1, IL8, and AGP concentrations in the uterus and the fornix were significantly higher in CC than healthy cows (CH). In conclusion, the 3-week postpartum is a critical point to evaluate cytokines and acute phase proteins; where IL1 and IL8 variation kept a direct relation with neutrophils numbers and function. The presence of AGP in the endometrium infer a homeostatic proinflammatory protective balance effect, modulating the local uterine innate immune response during peripartum.

## Introduction

Most parturient cows (90%) experience bacterial contamination of the uterine cavity and endometrial damages that trigger an active inflammatory response to clear the infection and repair the tissues, respectively (1, 2). However, the innate immune response does not always control uterine bacterial imbalances; therefore, causing a prolonged genital tract inflammation and a delay of the uterine involution indulging a postpartum uterine disease (3–6). The incidence of postpartum uterine disease (PUD) is high in dairy cows worldwide and includes clinical (metritis, endometritis) and non-clinically conditions such as subclinical endometritis (7) affecting the overall animal fertility.

The diagnosis of PUD is limited by the lack of clarity regarding the case criteria, difficulties associated with the diagnosis of subclinical conditions, and variable sensitivity and specificity of the different diagnostic tests in relation to the absence of direct visual assessment of the endometrium (8, 9). The discrepancies between clinical findings and the diagnostic test could be explained by the presence of an inflammatory process in tissues other than the endometrium like the cervix uteri and the vagina (10). The cytological evidence of cervical inflammation was reported in 19% of sub-fertile cows (11). Previous studies reported that 60.8 and 45% of dairy cows had clinical cervicitis and cytological cervicitis, respectively, between 42 and 50 days postpartum (10, 12). However, knowledge of inflammation or infection of the cervix and the subsequent influence on reproduction in dairy cows is limited.

The capacity of the uterus to resolve a genital tract infection depends on its ability to detect and respond to microbial ligands (1, 13). Innate immunity is the principal sensor responsible for immune response to control bacterial contamination of the uterus after parturition. The pathogen-associated molecular patterns (PAMPs) present in bacteria are quickly detected by the pattern recognition receptors (PRRs) in the host cells. The engage between PAMPs and PRRs such as Toll-Like Receptor (TLRs) initiate a signaling cascade resulting in the synthesis and production of pro-inflammatory cytokines such as Tumor Necrosis Factor alpha (TNF-α), interleukins (IL-1-α, IL-6) and chemokines (IL-8) (14, 15). For example, the chemokine IL8 is produced mainly by activated macrophages in response to bacterial PAMPs directing the upregulation of adhesion molecules to enhance neutrophil recruitment to the inflammatory area (13, 16). As a result, uterine inflammation is characterized by the significant presence polymorphonuclear leukocyte infiltration (Neutrophils) as well (14).

The lack of a complete genital examination in postpartum dairy cows without appreciation of the molecular immunological mechanisms underlying changes at the cellular or systemic level could explain the limited understanding of the general process involved in PUD (17). Therefore, the aim of the present study was to describe the innate immune response in the uterus (Ut) and vaginal fornix (Fx) in the pre- and post-partum periods of dairy cows with clinical cervicitis.

## Materials and Methods

### Study Population and Sampling

**Figure 1:**
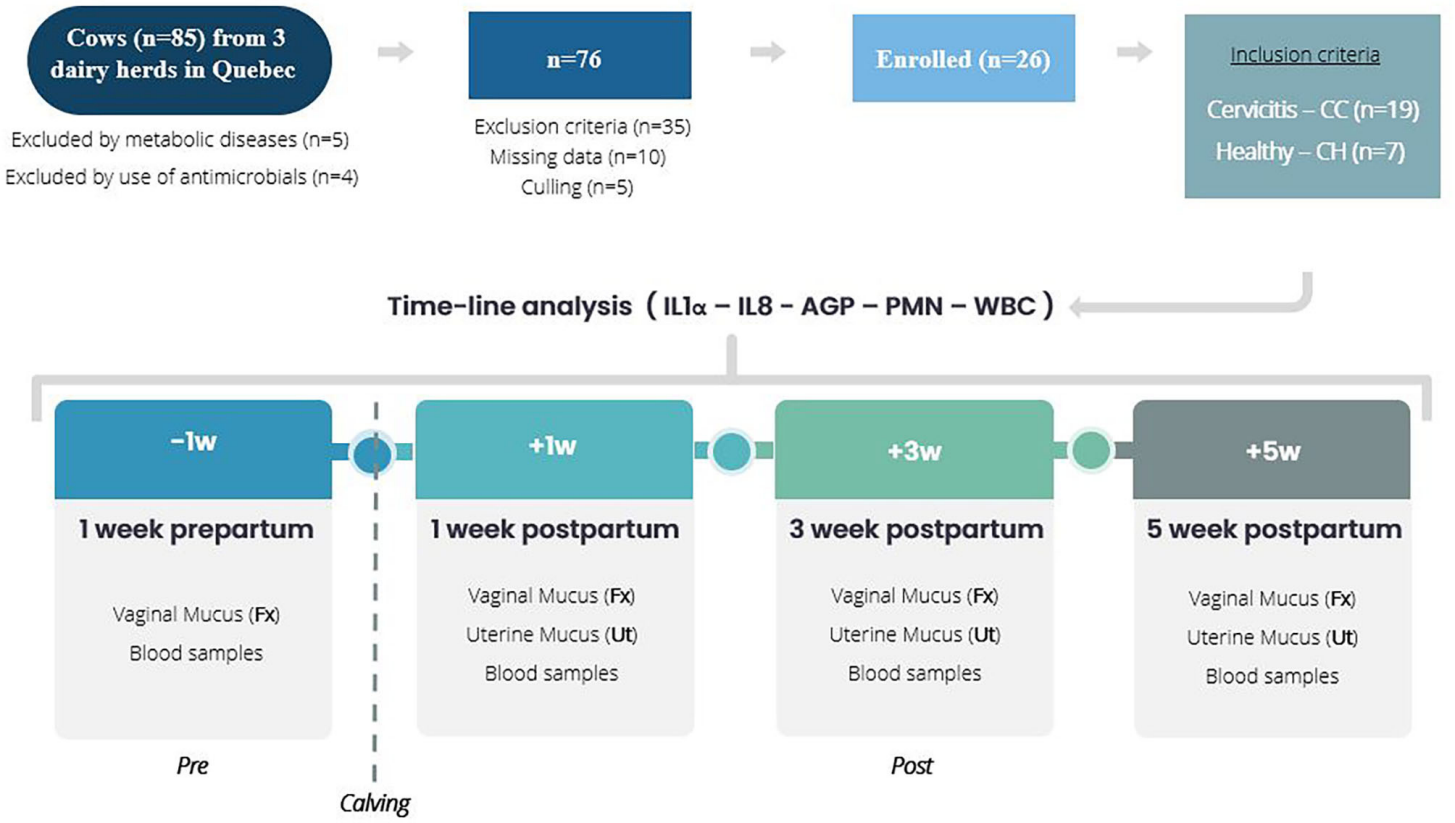
Study design. Timeline analysis of innate immune responses of clinical cervicitis in pre- and post-partum dairy cows (*n* = 26) from three dairy herds in Quebec in 2016. A retrospective study involving database and stored blood and mucus (vaginal fornix, Fx – uterine, Ut) sampling of 26 dairy cows from three commercial dairy herds in Quebec. Cows diagnosed with clinical cervicitis (*n* = 19) and healthy (*n* = 7) were involved in a time-course analysis from 1 week before calving to 5 weeks after calving to establish variations for white blood cell count (WBC), uterine PMN percentage, and vaginal fornix and uterine mucus cytokines (IL1 – IL8 – AGP).

A retrospective descriptive study (Figure 1) was performed involving 26 animals defined as, clinical cervicitis cows (*n* = 19) and healthy cows (*n* = 7). Cows were selected from a database of a previous sampling in which 85 dairy cows (purposively selected) were followed and sampled [blood and vaginal fornix (Fx) and uterine mucus (Ut)] during the pre- and postpartum period. For the present study, clinical data of the database and the stored blood and mucus samples were used. Blood samples were collected from the coccygeal vein of the tail head and placed in a tube with an anticoagulant, immediately placed on ice and transported to the laboratory within ~3 h. After 30 min at room temperature and gentle agitation, the complete hematology analysis was carried out with a VetScan HM5 hematology analyzer (Abaxis Global Diagnostics, Union City, CA, USA). The mucus and smears samples for cytokines and neutrophils percentage assessments were performed with the cytobrush technique (18). A sterile cytobrush (CytoSoft, Camarillo, CA, USA) was screwed onto a stainless steel rod (65 cm long ×4 mm diameter) within a stainless steel tube. The apparatus was inserted into a hard protective plastic sheath (IMV Technologies, L'Aigle, France) before insertion into a second protective sheath (30 cm long Sani-Shield Rod Protector, Agtech, Manhattan, NY, USA). The doubled sheathed instrument was used to collect cellular material of mucosa of vaginal fornix and endometrium using the sterile cytobrush. A cervical sample brush and uterine sample brush were collected into a sterile tube with 1 ml of phosphate buffered saline (PBS) and stored frozen at −80°C and used in the present study to determine the immune response.

The cows came from three commercial dairy herds in Québec (Canada) that were housed in a tie-stall barn and milked twice daily. The rolling herd average for milk production was 9,000 kg. Cows were fed a total mixed ration of corn and hay silage to meet the nutrient requirements of dairy cows recommended by the National Research Council (19). Farms were visited weekly by the same veterinarians and all cows were vaccinated at 40 and 26 days before parturition against E. coli (2 ml IM, J-VAC®, Boehringer Ingelheim, Burlington, Ontario, Canada, L7L 5H4) and between 10 and 30 days postpartum against IBR, BVD type 1, BVD type 2, PI3, and BRSV (2 ml IM, Bovi-shield GOLD5® FPTM 5 L5, Zoetis, Kirkland, Québec, Canada, H9H 4M7). In addition, all pregnant cows were injected with Se (5.0 ml, D-60 before calving, MU-SE, Intervet, Merk, Kirkland, Québec, Canada, H9H 4M7).

The database includes records of cow's clinical exam performed four times: −1 w (day −7 ± 2, prepartum), +1 w (day +7 ± 4), +3 w (day +21 ± 4) and +5 w (day +35 ± 4) postpartum (Figure 1). Data collection included clinical data associated with reproductive assessment (vaginoscopy, transrectal exams, purulent vaginal discharge, cervix characteristics, uterine horn symmetry, and ovarian structures), cytological exams (neutrophil count in the fornix of the vagina and in the uterus) and blood samples (white blood cell count).

### Case Definition

For the animal selection, postpartum disorders were defined. Clinical metritis was characterized as abnormally enlarged uterus and purulent uterine discharge within the first 21 days postpartum without systemic clinical sings (20). At 5 weeks postpartum, clinical endometritis (CE) was defined as a cow exhibiting purulent vaginal discharge – PVD (20) or subclinical endometritis (SE) in cows with a proportion of neutrophils exceeding 5% on endometrial cytology in absence of PVD and anomalies on transrectal examination (9). On vaginoscopy, cervix was classified as GRADE 0 (Normal) without abnormality; GRADE 1 (normal) with the second cervical fold swollen without redness and prolapsing through the first ring, and GRADE 2 (Clinical Cervicitis, CC) with the second fold swollen and red prolapsing through the first ring without PVD (10).

### Exclusion Criteria and Animal Selection

Based on reproductive assessment records, as exclusion criteria, animals were rejected of the database because of: (1) systemic illness or clinical conditions other of the reproductive tract (2) calving disorders (dystocia, twins, fetal membranes retention) and (3) animals that in the final reproductive exam (+5 w), exhibited endometritis (clinical or subclinical) without clinical evidence of clinical cervicitis. Clinical cervicitis (CC) criteria in this research was considered when, posterior to applying the exclusion criterion, the cervix had reddening of the supra-vaginal portion of the cervix with edema and prolapse of the second cervical fold (cervix grade−2 at the 5-week examination). Healthy cows (CH) were defined as a cow clinically normal with a cervix grade-0 or grade-1 at the 5 w and, without postpartum disorders during all the follow-up period (−1 w +1 w; +3 w; +5 w postpartum).

From the data (*n* = 85), animals were rejected because of culling (*n* = 5), use of antimicrobials (*n* = 4), metabolic disease (*n* = 5), missing data (*n* = 10), and exclusion criteria (*n* = 35). Finally, 26 cows were enrolled in the study: 19 met the case definition criteria for cervicitis and seven met the criterion defined for healthy cows (Figure 1).

### Measurement of Cytokine Concentration in Vaginal and Uterine Samples

The concentrations of interleukin 1α (IL1), interleukin 8 (IL8), and α1-acid glycoprotein (AGP) were determined by using commercial kits (GENORISE SCIENTIFIC, INC. Philadelphia, USA): Bovine IL-1α ELISA Kit Nori® (minimum detection range 3–200 pg/ml, assay sensitivity 1 pg/ml, intra-assay CV 6%, inter-assay CV 9%); Bovine IL-8 ELISA Kit Nori® (minimum detection range 12–800 pg/ml, assay sensitivity 2 pg/ml, intra-assay CV 6%, inter-assay CV 9%) and; Bovine AGP ELISA Kit Nori® (minimum detection range 2.5–160 ng/ml, assay sensitivity 0.5 ng/ml, intra-assay CV 5%, inter-assay CV 9%). All procedures were performed according to the guidelines provided by the manufacturers. Briefly, centrifugation of the content of stored tubes (cytobrush with cervical or uterine mucus in 1 ml PBS) was performed to collect the supernatant of uterine mucus and vaginal fornix mucus before to be added to 96-well microplates and incubated for 1 h at room temperature. After aspiration and washing (with 300 μL of Assay Buffer, three times), 100 μL of the working dilution of Detection Antibody (diluted in Regent Diluent) were added to each well and incubated for 1 h. The plates were washed and then incubated with 100 μL of the working dilution of HRP conjugate solutions for 20 min at room temperature, washed again, and then incubated with 100 μL of the substrate solution for 5 min. Finally, 50 μL of the Stop Solution were added. The optical density of each well was then measured at 450 nm in Microplate Spectrophotometer (SpectraMax® Plus 384 Absorbance Plate Reader, USA). A standard curve for each ELISA Kit was constructed by plotting the mean absorbance for each standard on the y-axis against the concentration on the x-axis to calculate the R^2^ coefficient.

### Data Analysis

Categorical variables included in the analysis were time of examination (−1, +1, +3, +5 w weeks postpartum), sample origin (Vaginal fornix – Uterus) and clinical profile [healthy (CH), clinical cervicitis (CC), clinical cervicitis + purulent vaginal discharge (CC + PVD), clinical cervicitis + subclinical endometritis (CC + SE)]. Continuous variables were concentrations of IL1, IL8, and AGP, the proportion of polymorphonuclear neutrophils (PMN) in the vaginal fornix and the uterus (percentage of PMN in a 300-cell count), and the white blood cell count (WBC).

Continuous variables were assessed for normal distribution using histogram with Gaussian distribution graph and Shapiro-Wilk test. A nonparametric test (Mann-Whitney) was used to estimate a difference in the concentrations of IL1, IL8, AGP into the comparisons groups: (1) CC – CH; (2) Ut – Fx and (3) (−1 w) – (+1 w) – (+3 w) – (+5 w). When a significant difference was found, the power to detect a difference (with a 95% confidence interval) was calculated considering the number of cows per group (cervicitis – Healthy) and the mean and standard error of the mean (for IL1, IL8, AGP) found in each comparisons group. The statistical differences found were reported only when the power was ≥70%.

All analyses were conducted in Stata® Statistical Software (Release 15. College Station, TX: Stata Corp LP).

## Results

### Clinical Characteristics of Cervicitis

This research showed that 23% of the cows (*n* = 19) at +5 w were categorized as cervicitis. In addition, some cases of CC appeared with purulent vaginal discharge – CC + PVD (*n* = 4) or subclinical endometritis – CC+SE (*n* = 9).

### Percentage of PMN in the Mucus of the Vaginal Fornix and the Uterus

**Figure 2:**
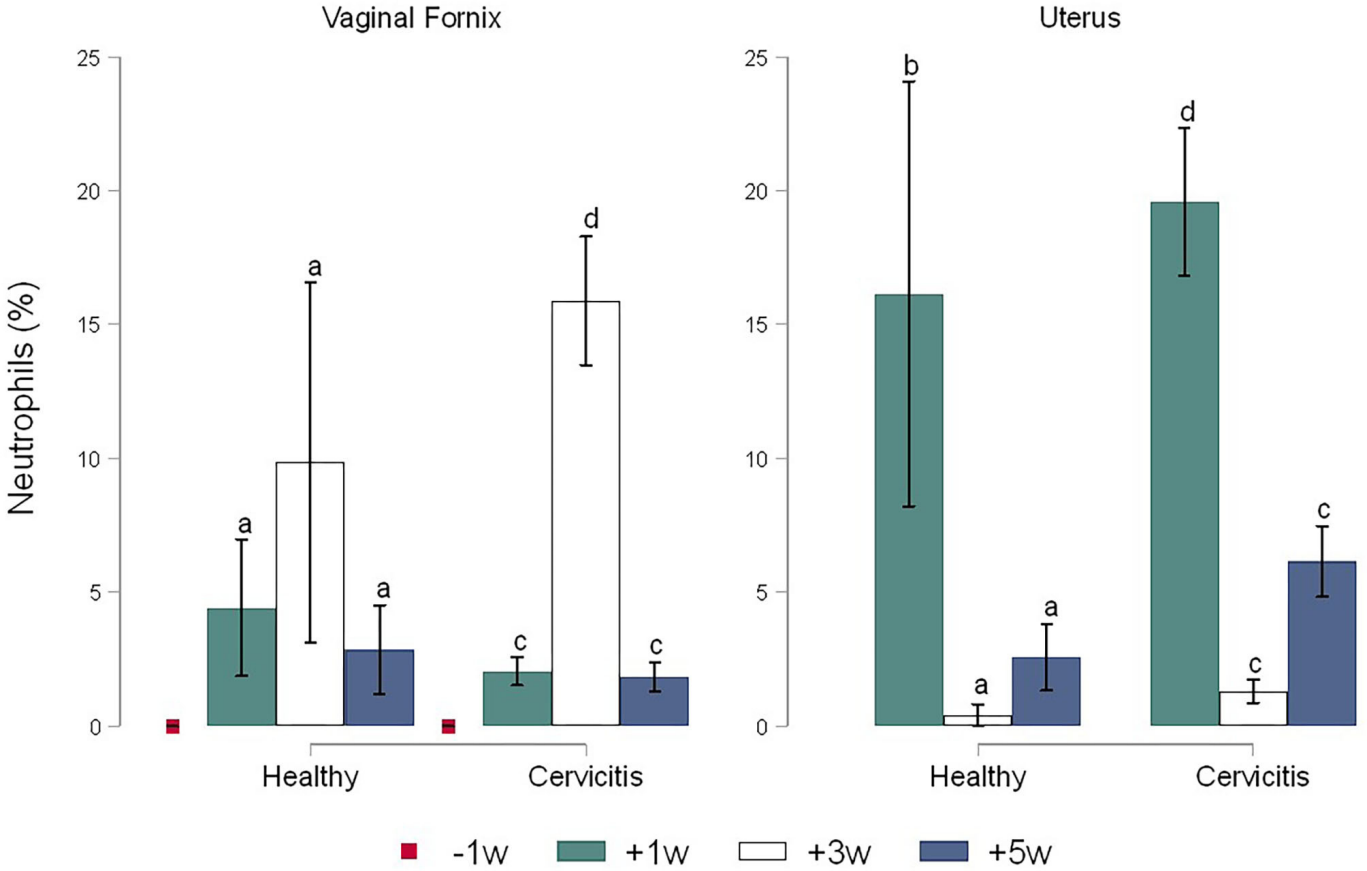
Time-course variations in the pre-and post-partum percentage of neutrophils in the vaginal fornix and uterine mucus of cows (*n* = 26) defined as clinical cervicitis cows (*n* = 19) and healthy cows (*n* = 7) from three dairy herds in Quebec in 2016. Time-course variations (weeks −1 w +1 w +3 w +5 w) of neutrophils percentage, time-course differences are indicated with superscript a-b (for healthy cows), and c-d (for clinical cervicitis cows). Means without a common superscript differed (*P* < 0.05).

The percentage of PMN in the vaginal fornix of CC cows changed during the sampling time pre- and post-partum respectively. On time +3 w, PMN in the vaginal fornix of CC cows were 15.89% (*P* = 0.001), 13.84% (*P* = 0.005) and 14.05% (*P* = 0.004) higher than at −1, +1, and +5 w respectively. There were not significant differences between CH and CC animals at −1 w however, there were significant differences between CH and CC animals (*p* < 0.05) at +1, +3, and +5 w respectively. In CC cows, the PMN% was the highest at +3 w and the lowest at +1 and +5 w. In uterine mucus, cows with CC showed a significant difference (*p* < 0.05) in the PMN% in all the sampling times with the highest percentage at +1 w. However, there was no difference between CC/CH (Figure 2).

### Cytokine Variations by Examination Time, Case/Control, and Sample Origin

**Figure 3:**
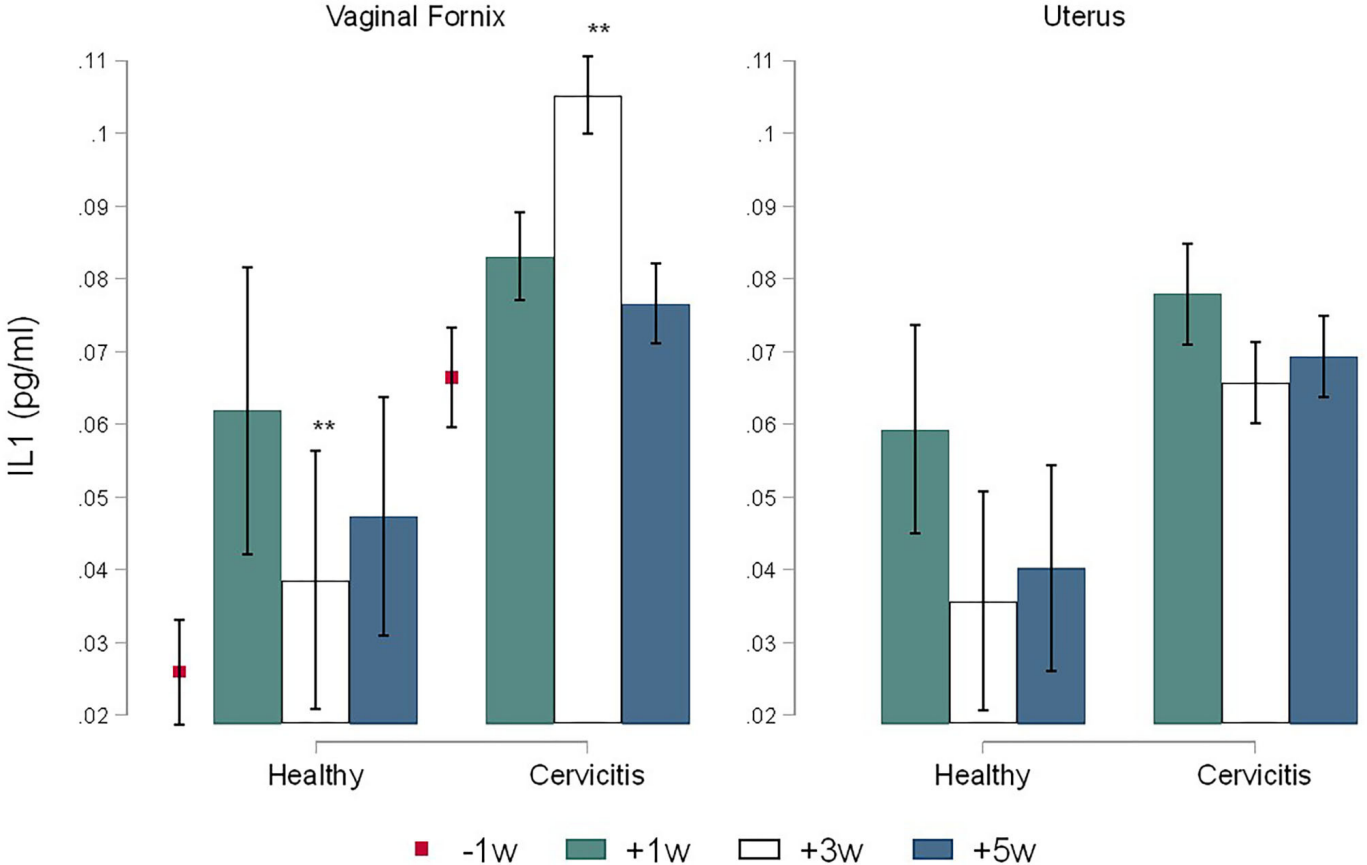
Time-course variations (Mean ± SEM) in pre and post-partum concentrations of IL1 IL8 in the vaginal fornix and uterine mucus of cows (*n* = 26) defined as clinical cervicitis cows (*n* = 19) and healthy cows (*n* = 7) from three dairy herds in Quebec in 2016. Time-course variations (weeks −1 w +1 w +3 w +5 w) of Interleukin 1α (pg/ml) concentrations (Mean ± SEM). An asterisk indicates a significant difference at *p* ≤ 0.05 (*) or *p* ≤ 0.01 (**) for the Mann-Whitney Test (Ha: clinical cervicitis cows ≠ healthy cows).

**Figure 4:**
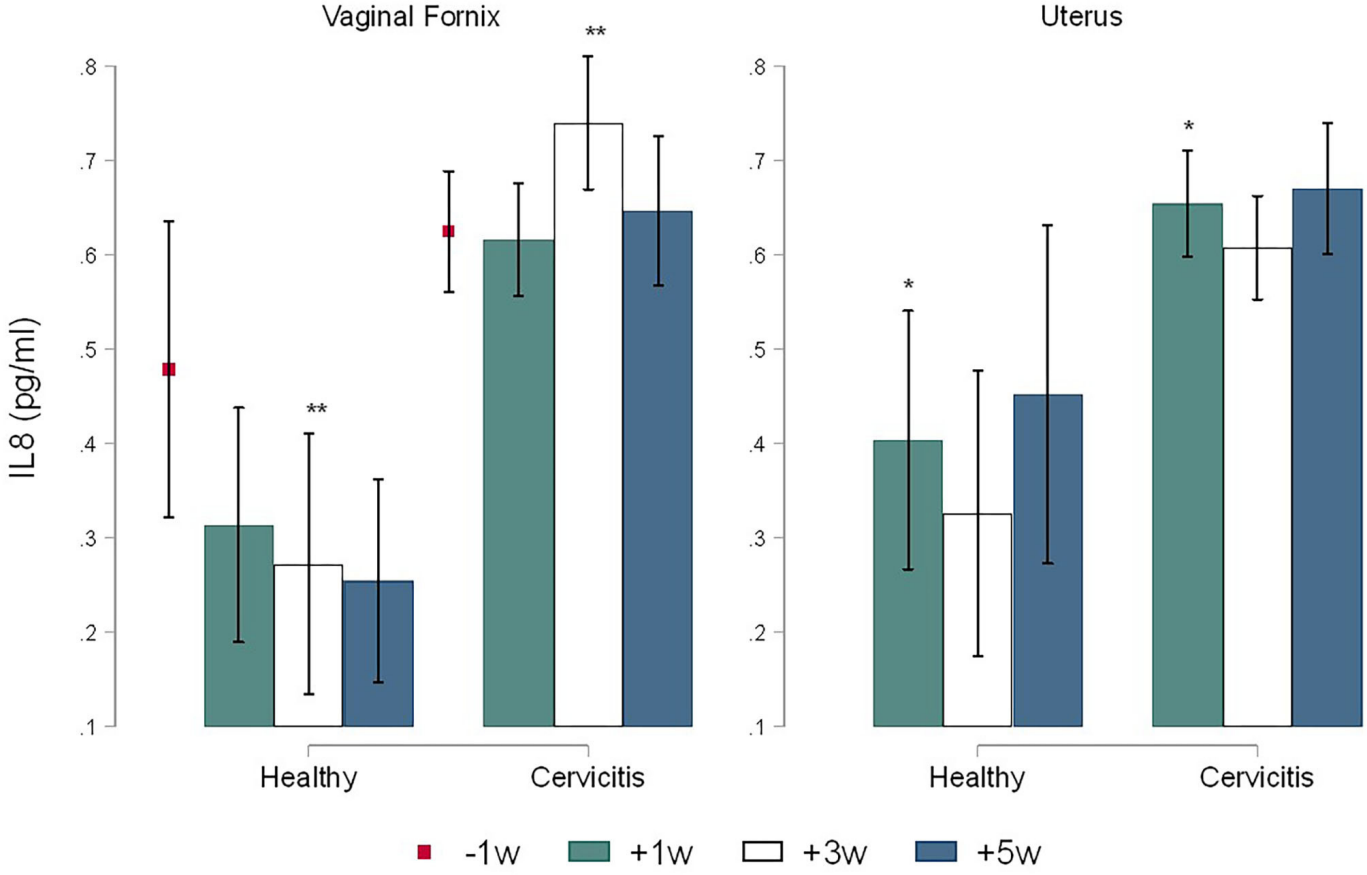
Time-course variations (Mean ± SEM) in pre and post-partum concentrations of IL8 in the vaginal fornix and uterine mucus of cows (*n* = 26) defined as clinical cervicitis cows (*n* = 19) and healthy cows (*n* = 7) from three dairy herds in Quebec in 2016. Time-course variations (weeks −1 w +1 w +3 w +5 w) of α1-acid glycoprotein (pg/ml) concentrations (Mean ± SEM). An asterisk indicates a significant difference at *p* ≤ 0.05 (*) or *p* ≤ 0.01 (**) for the Mann-Whitney Test (Ha: clinical cervicitis cows ≠ healthy cows).

**Figure 5:**
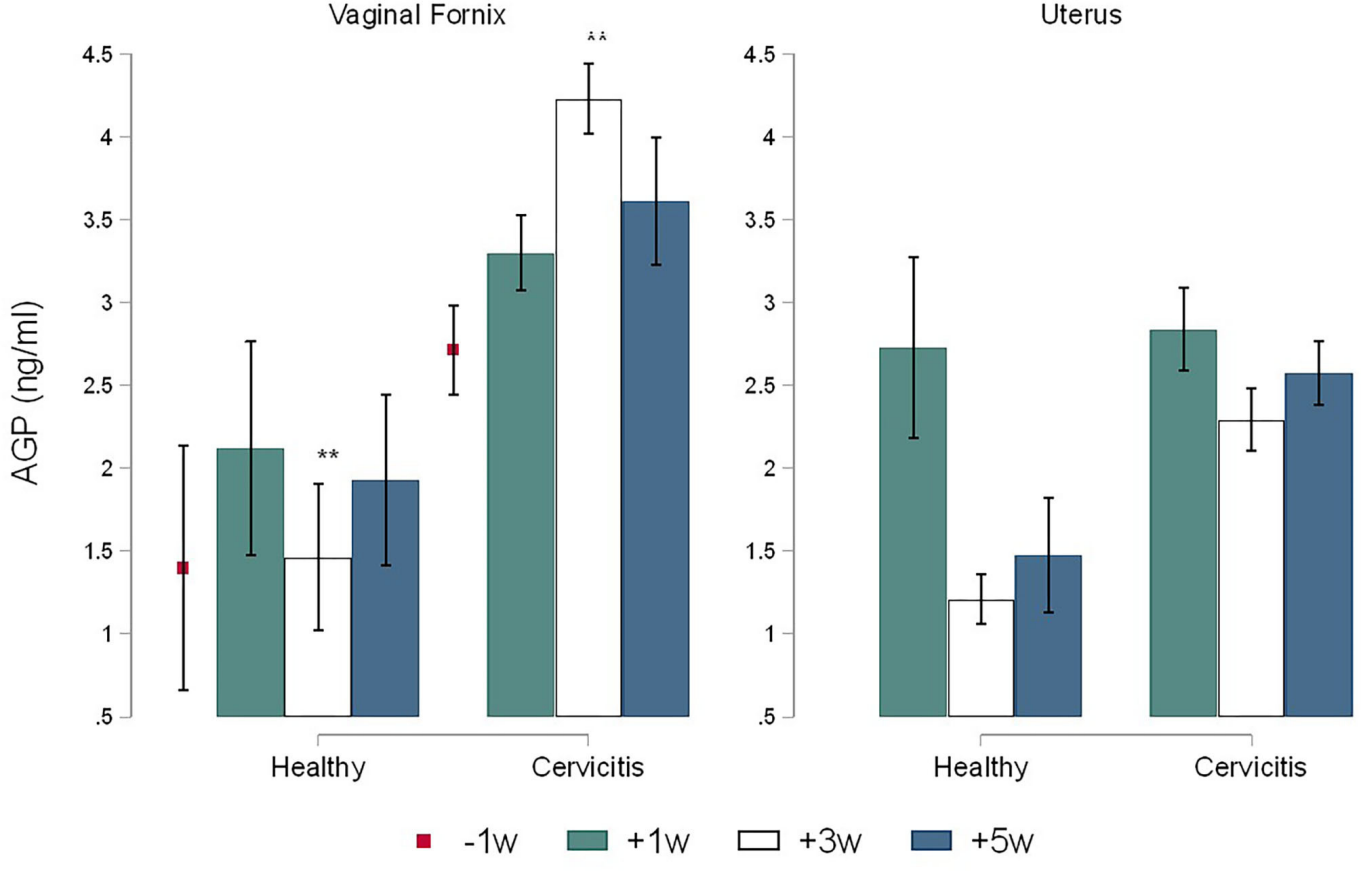
Time-course variations (Mean ± SEM) in pre-and post-partum concentrations of AGP in the vaginal fornix and uterine mucus of cows (*n* = 26) defined as clinical cervicitis cows (*n* = 19) and healthy cows (*n* = 7) from three dairy herds in Quebec in 2016. Time-course variations (weeks −1 w +1 w +3 w +5 w) of α1-acid glycoprotein (ng/ml) concentrations (Mean ± SEM). An asterisk indicates a significant difference at *p* ≤ 0.05 (*) or *p* ≤ 0.01 (**) for the Mann-Whitney Test (Ha: clinical cervicitis cows ≠ healthy cows).

The calculate *R*^2^ coefficient for IL1, IL8, and AGP were 0.75, 0.86, and 0.98 respectively. No significant differences were found for cytokines by sampling times. Comparison between CC and CH cows showed statistical differences only for time +3 w where the concentrations of IL1 in Fx (1.05 ± 0.01 pg/ml) and in Ut (0.065 ± 0.01 pg/ml) of CC cows were significantly higher (*P* = 0.005) than in the Fx (0.038 ± 0.019 pg/ml) and in the Ut (0.035 ± 0.016 pg/ml) of CH cows (Figure 3). In addition, the concentrations of IL8 in CC cows were more elevated than CH cows in both the Fx (0.73 ± 0.14 pg/ml vs. 0.27 ± 0.14 pg/ml, *P* = 0.043) and uterine (0.60 ± 0.11 pg/ml vs. 0.32 ± 0.16 pg/ml, *P* = 0.040) at +3 w respectively (Figure 4). The concentration of AGP in Fx (Figure 5) for CC was 2.76 ng/ml higher than CH (*P* = 0.0018) at +3 w. Comparison between Fx and Ut for CC cows at +3 w showed that IL1 and AGP concentrations in Fx were 0.003 ng/ml (*P* = 0.018) and 1.93 ng/ml (*P* = 0.002) higher respectively than in the Ut samples. No significant difference was found between Fx and Ut concentrations of IL1, IL8 and AGP in CH cows at time +3 w (*p* > 0.05).

### Cytokine Variations by Clinical Characteristics of Cervicitis

Cases of cervicitis (CC) diagnosed at +5 w occurred concurrently with other uterine disorders (Table 1). At the time −1 w, Fx IL1 was higher in cows with CC+SE compared with CH cows (*P* = 0.016). At the time +3 w, Fx IL1 and IL8 concentrations were higher for CC+SE cows compared with CH cows (*P* < 0.05). At the time +5 w, Fx IL8 and AGP were higher for CC + SE cows compared with CH cows (*P* < 0.05). In uterine samples, at time +3 w: (a) Ut IL1 was higher in cows with CC + SE compared with CC + PVD cows (*P* = 0.08); (b) Ut AGP was higher for CC + SE cows compared with CC + PVD cows (*P* = 0.028).

**Table 1:** Mean ± SEM for AGP, IL1, and IL8 in vaginal fornix and uterine mucus of pre-and post-partum cows (*n* = 26) defined as healthy, clinical cervicitis, and clinical cervicitis plus additional uterine disease conditions.

**Week**	**Sample**	**Disease**	**IL1 (pg/ml)**	**IL8 (pg/ml)**	**AGP (ng/ml)**
			**M ± SEM**	**M ± SEM**	**M ± SEM**
**–**1 w	Fx	CH	0.02 ± 0.00	0.47 ± 0.16	1.39 ± 0.79
		CC	0.06 ± 0.02	0.55 ± 0.18	2.51 ± 0.72
		CC + PVD	0.03 ± 0.01	0.39 ± 0.13	1.82 ± 0.54
		CC + SE	0.12 ± 0.03^a^	1.24 ± 0.37	5.02 ± 1.8
+1 w	Fx	CH	0.06 ± 0.02	0.31 ± 0.13	2.11 ± 0.69
		CC	0.07 ± 0.02	0.53 ± 0.21	3.38 ± 0.65
		CC + PVD	0.07 ± 0.01	0.50 ± 0.15	3.22 ± 0.88
		CC + SE	0.10 ± 0.02	0.98 ± 0.33	3.33 ± 0.77
	Ut	CH	0.05 ± 0.01	0.40 ± 0.14	2.73 ± 0.58
		CC	0.06 ± 0.02	0.48 ± 0.18	2.32 ± 0.87
		CC + PVD	0.06 ± 0.01	0.54 ± 0.10	2.63 ± 0.73
		CC + SE	0.12 ± 0.04	1.16 ± 0.34	4.05 ± 1.40
+3 w	Fx	CH	0.03 ± 0.01	0.27 ± 0.14	1.46 ± 0.48
		CC	0.09 ± 0.01	0.58 ± 0.21	3.19 ± 0.31
		CC + PVD	0.09 ± 0.01	0.55 ± 0.16	4.64 ± 0.77
		CC + SE	0.13 ± 0.03^a^	1.37 ± 0.39^a^	4.85 ± 0.95
	Ut	CH	0.03 ± 0.01	0.32 ± 0.16	1.21 ± 0.16
		CC	0.05 ± 0.02	0.52 ± 0.16	1.83 ± 0.63
		CC + PVD	0.05 ± 0.01	0.45 ± 0.13	1.85 ± 0.50
		CC + SE	0.11 ± 0.01^b^	1.08 ± 0.31	3.96 ± 0.74^b^
+5 w	Fx	CH	0.04 ± 0.01	0.25 ± 0.11	1.93 ± 0.55
		CC	0.07 ± 0.02	0.48 ± 0.21	2.37 ± 0.64
		CC + PVD	0.06 ± 0.01	0.41 ± 0.14	2.70 ± 0.52
		CC + SE	0.10 ± 0.02	1.40 ± 0.52^a^	7.5 ± 3.00^a^
	Ut	CH	0.04 ± 0.01	0.45 ± 0.19	1.47 ± 0.37
		CC	0.07 ± 0.01	0.55 ± 0.23	2.16 ± 0.54
		CC + PVD	0.04 ± 0.01	0.47 ± 0.14	2.37 ± 0.54
		CC + SE	0.11 ± 0.02	1.27 ± 0.38	3.64 ± 1.23

Time-course variations (weeks **–**1 w +1 w +3 w +5 w) of Interleukin 1α (pg/ml), Interleukin 8 (pg/ml) and α1-acid glycoprotein (ng/ml) concentrations (Mean ± SEM) in vaginal fornix (Fx) and uterine mucus (Ut) of cows with cervicitis CC, cervicitis plus purulent vaginal discharge (CC + PVD), cervicitis plus subclinical endometritis (CC + SE), and healthy CH.

a Indicate a significative difference Mann-Whitney Test p ≤ 0.05 (compared with CH cows).

b *Indicate significative difference Mann-Whitney Test p ≤ 0.05 (compared with CC + PVD cows)*.

### White Blood Cell Count (WBC), Variations by Examination Time and Case/Control

**Figure 6:**
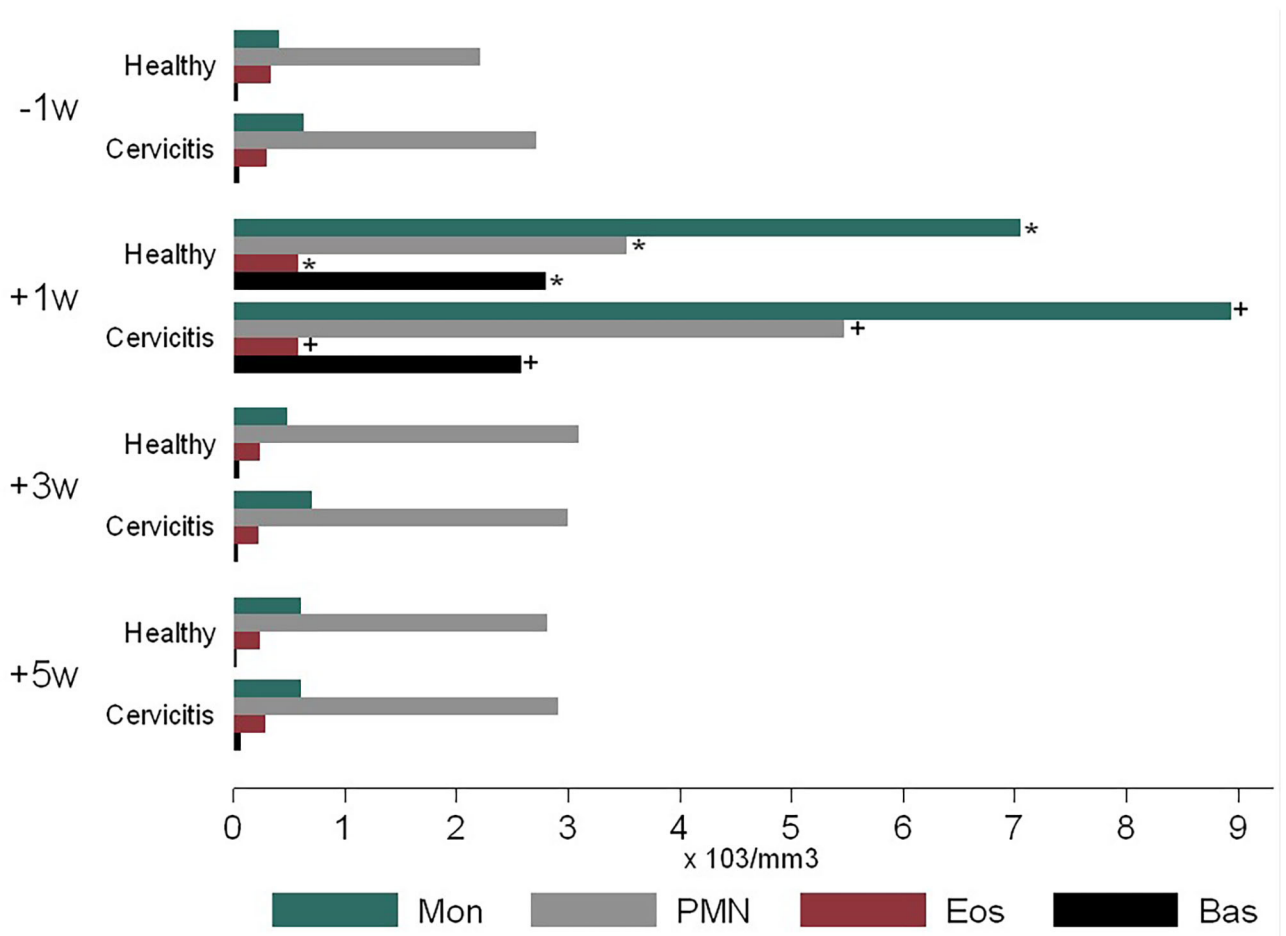
Time-course variations in pre-and post-partum white blood cell count of cows (*n* = 26) defined as clinical cervicitis cows (*n* = 19) and healthy cows (*n* = 7) from three dairy herds in Quebec in 2016. Time-course variations (weeks −1 w +1 w +3 w +5 w) of blood cell count (× 103 mm^3^) for monocytes (Mo), Neutrophils (Neu), Eosinophils (Eos), Basophiles (Bas). Body mark (^*^) indicates a significative difference in CH cows at *p* ≤ 0.05 for sample time comparison (weeks −1 w +1 w +3 w +5 w). Body mark (+) indicates a significative difference in CC cows at *p* ≤ 0.05 for sample time comparison (weeks −1 w +1 w +3 w +5 w).

For monocytes (Mon), neutrophils (PMN) eosinophils (Eos), and basophils (Bas), there were no significant differences (*p* > 0.05) between CC cows and healthy cows (CH). At the time +1 w, WBC was higher compared to −1, +3, and +5 w (Figure 6).

## Discussion

Innate immunity plays an important role in keeping postpartum reproductive tract microflora balance; where the cervix acts as an anatomical barrier protecting the uterus from external pathogens producing cervical mucus (12, 21, 22). During postpartum, puerperal physiological modifications occurring in the reproductive tract, changes in the local microflora and arisen of potential pathogens, and physical traumas associated with calving or obstetrical manipulations may trigger cervical inflammation. In the present study, clinical cervicitis was diagnosed in 23% of the cows examined at 5 weeks postpartum showing a red, edematous, and prolapsed cervical fold. Moreover, some cases were accompanied by purulent vaginal discharge or subclinical endometritis. The authors measured an increase of neutrophils in the reproductive tract (uterus and fornix), a major production of pro-inflammatory cytokines (IL-1) and chemokines (IL-8), and the recruitment of circulating neutrophils and monocytes meaning that postpartum cervicitis is a process initially controlled by innate immunity. In addition, cervicitis was accompanied by uterine inflammation (clinical and subclinical endometritis) in 41% (*n* = 13) of the cases. Interaction between clinical endometritis, subclinical endometritis, and cervicitis is not well-characterized. However, even with a clear physical partition of the reproductive tract and a well-defined histologic definition of each organ in cows one can hypothesize that the process of clearance of the reproductive tract inflammation in postpartum cows may proceed from the upper (uterus) to the lower organs (cervix). More studies are needed to understand the process of inflammation and the interaction between the different compartments of the reproductive tract of the cow in a postpartum situation.

The current research describes the innate immune response in the uterus and the vaginal fornix, and the variations occurring during the pre- and postpartum periods (−1, +1, +3, +5 w) in cows with clinical cervicitis. The concentrations of IL-1, IL8, and AGP in the Fx mucus of CC cows increased from −1 to +3 w reaching the highest concentration at +3 weeks followed by a decrease at +5 w. However, the concentrations of cytokines and the chemokine stayed high during the whole postpartum period (+1 to +5 w) in CC cows. The present results correlate with the increase of the bacterial community in the vagina most likely coming from the environment in the weeks after calving (20, 23). The postpartum period is characterized by calving-associated physical barriers relaxation including an open cervix and a negative pressure created by repeated uterine contraction and relaxation. As a result, there is an enhancement of bacterial contamination in bovine uteri and vagina after calving, initially by Gram-negative bacteria follow by Gram-positive bacteria decreasing the levels of neutrophils and cytokines (2). Therefore, inflammatory cytokines are expressed in the vaginal and uterine mucosa in a time-related manner during the postpartum period with a significant increase around 3 weeks after calving (24). As partitioning of the reproductive tract is reestablished with closure of the cervix and bacteria trapped in the reproductive tract, the innate immune response may persist longer in CC cows comparatively to CH cows where bacterial clearance is more prompt.

In the current research, the results of CC cows show an increased concentration of AGP in relation to IL1 and IL8 in Fx. The α1-acid alpha glycoprotein AGP is an Acute Phase Protein normally expressed by the liver and usually found in blood, however extrahepatic expression of bovine AGP has been reported in tissues like uteri and ovaries (25–29). The protein AGP possesses an immunomodulatory activity and it is believed to play an important role in the regulation of local inflammation by reducing the tissue damages caused by excessive activation of the complement. It modulates apoptosis in bovine monocytes and the degranulation of neutrophils involved in the fine-tuning of neutrophil activity during the inflammation (27, 28, 30, 31). In the study, AGP concentrations (Figure 5) increased during cervicitis, mainly at +3 w (*p* < 0.05) however, when cervicitis and subclinical endometritis were both present AGP concentration in Fx was higher at 5 w (*p* < 0.05) (Table 1). The present results agree with previous work that demonstrated a higher level of plasma AGP in cows developing uterine infection in comparison with cows without endometritis (32). AGP can modulate locally the uterine innate immune response in different cases such as normal uterine involution, cervicitis or endometritis without the presence of a systemic inflammatory process. In cows, AGP is contained in neutrophil granules and released when activated. More specifically, the highly glycosylated AGP is syntheses in myelocytes, stored, and released in secondary granules by activated neutrophils representing a fine-tuning of the neutrophil's function (Down-regulatory effect) reducing the damages caused by an excess of inflammatory response (28, 31, 33). In vaginal samples of CH, similar concentrations of AGP (Figure 5) were found during the pre- and the postpartum period in concordance with the changes in neutrophils (Figure 2) indicating some endogenous homeostatic role. The present results infer that endometrial and vaginal production of AGP has an important role in neutrophil function in both in healthy and infected cows. However, there is scarce information related with the role of AGP in postpartum uterine disease in dairy cows and, a potential use of acute phase proteins as predictor of postpartum uterine infections in dairy cows have been reported (34), therefore more research is needed.

The parturition period requires extensive remodeling of the cervix before parturition (cervical softening and ripening) for the transformation of the cervix from a closed rigid structure to one that opens for birth (35, 36). At −1 w, a high blood neutrophil count, an increased concentration of IL-8, and a detectable IL1 and AGP concentrations were measured in CC and CH cows. The present results agree with the increase in expression of IL-8 enabling the influx of neutrophils in the cervical tissue that excretes the matrix metalloproteinase which contributes to the softening of the cervix (37, 38). Moreover, parturition is associated with the local accumulation of IL8, which acts synergistically with PGE2 to attract polymorphonuclear neutrophils. Hence, activation of placental leukocyte population releases proteolytic enzymes that degrade the caruncles, thus facilitating fetal membrane separation and advancing the progression of labor (37, 39). Therefore, fetal membrane separation is directly linked to inflammatory changes in the uteroplacental interface. The current results from −1 w prepartum to +1 w postpartum might explain the link between the increase of uterine IL-8, the blood monocytes levels, and the endometrial concentrations of neutrophils. Increased apoptosis, caruncular degradation, and IL-8 production around parturition trigger a massive influx of neutrophil and mononuclear cells into the uterus and the cervix that expel their placenta normally (39). The prepartum increased IL8 concentration found in the present research could be related to cervical ripening before parturition which is a physiological pro-inflammatory process influenced by regulatory cytokines.

In the first week postpartum, the current research found an increase in uterine neutrophils percentage, blood neutrophils and monocytes levels (Figures 2–6). This is an expected finding considering that tissue macrophages release IL1 and IL8 acting as a pro-inflammatory and chemo-attracting process coordinating the recruitment of neutrophils to the site of infection enhancing an inflammatory repairing response. The increase of blood monocytes at +1 w (Figure 6) may result in more differentiation of monocyte in macrophages in the reproductive tissues for a better clearance of pathogens and a more effective control of inflammation (15, 40). The abundance of monocyte around parturition reflects the participation of macrophages in the recognition and phagocytosis of cells undergoing apoptosis and capture of bacterial lipopolysaccharides (41). The present results agree with the important role of the expression of pro-inflammatory cytokines and chemokines for calving; where IL1 and IL8 concentrations were at least 8-fold higher in the cervix at parturition than during the gestation (38).

In conclusion, the present study describes cytokines and AGP modulation of the local innate immune response in cows with cervicitis during the postpartum period. The clinical findings of the present study allow diagnosing the presence of a regulated proinflammatory process in the external section of the cervix with certain reliability. The prolapse of the second cervical fold could be related to an inflammatory process in the inner part of the cervix also (clinical cervicitis). The 3 w postpartum was found to be a critical point showing significative increase in the concentration for all markers of inflammation studied. Variations of IL1 and IL8 during the time course of the study were associated with the neutrophils function and most likely part of a normal homeostatic process. These cytokines concentrations were higher in cows with clinical cervicitis than healthy cows. With a proinflammatory protective effect, IL1, IL8 and AGP can modulate locally the uterine innate immune response involving neutrophils in normal uterine involution and in cases of cervicitis or endometritis without the presence of a systemic inflammatory process. Cervicitis can occur in the concomitance of other recognized postpartum uterine clinical conditions (clinical - subclinical endometritis) potentially reducing fertility. As a limitation of the study, we found concentrations for IL1 and IL8 below of the detection range reported for the kit, this is related to the type of sample used [brush with cervical mucus sample and brush with uterine mucus sample collected in a sterile tube with 1 ml of phosphate-buffered saline (PBS) and stored frozen at −80°C]. The kits were selected based on the manufacturer's recommendations for the type of sample. Nevertheless, considering that studies on this topic are scarce, and have different disease definitions, research methods, and limitations (small number of observations, retrospective data, the absence of a sample size calculation, discrepancies between range detection reported for the kits and IL concentrations), further information is needed to understand better the impact of cervicitis on postpartum uterine disease and fertility.

## Data Availability Statement

The raw data supporting the conclusions of this article will be made available by the authors, without undue reservation.

## Ethics Statement

This research was performed in compliance with the experimental practices and standards approved by the animal care committee of the University of Montreal (247-29-227).

## Author Contributions

DV-T and RL conceived and designed the study and analyzed the data and performed the statistical analysis. AB, NP-C, and RL collected clinical data and mucus and blood samples. DV-T conducted the cytokine assays. DV-T, AB, MS, and RL organized and interpreted the database. DV-T, MS, and RL wrote and revised the manuscript. All authors contributed to the article and approved the submitted version.

## Conflict of Interest

The authors declare that the research was conducted in the absence of any commercial or financial relationships that could be construed as a potential conflict of interest.
